# Implementing and validating a home-infusion central-line–associated bloodstream infection surveillance definition

**DOI:** 10.1017/ice.2023.70

**Published:** 2023-11

**Authors:** Sara C. Keller, Susan M. Hannum, Kimberly Weems, Opeyemi Oladapo-Shittu, Alejandra B. Salinas, Jill A. Marsteller, Ayse P. Gurses, Eili Y. Klein, Ilya Shpitser, Christopher J. Crnich, Nitin Bhanot, Clare Rock, Sara E. Cosgrove, the Home Infusion CLABSI Prevention Collaborative

**Affiliations:** 1 Division of Infectious Diseases, Department of Medicine, Johns Hopkins University School of Medicine, Baltimore, Maryland; 2 Department of Health Policy & Management, Johns Hopkins Bloomberg School of Public Health, Baltimore, Maryland; 3 Armstrong Institute of Patient Safety and Quality, Johns Hopkins Medicine, Baltimore, Maryland; 4 Department of Health Behavior and Society, Johns Hopkins Bloomberg School of Public Health, Baltimore, Maryland; 5 Department of Hospital Epidemiology and Infection Control, Johns Hopkins Health System, Baltimore, Maryland; 6 Department of Infection Prevention, Nuvance Health Vassar Brothers Medical Center, Poughkeepsie, New York; 7 Department of Emergency Medicine, Johns Hopkins University School of Medicine, Baltimore, Maryland; 8 Malone Center for Engineering in Health Care, Johns Hopkins Whiting School of Engineering, Baltimore, Maryland; 9 Department of Computer Science, Johns Hopkins Whiting School of Engineering, Baltimore, Maryland; 10 Division of Infectious Diseases, Department of Medicine, University of Wisconsin School of Medicine, Madison, Wisconsin; 11 Division of Infectious Diseases, Department of Medicine, Allegheny Health Network, Pittsburgh, Pennsylvania

## Abstract

**Objective:**

Central-line–associated bloodstream infection (CLABSI) surveillance in home infusion therapy is necessary to track efforts to reduce infections, but a standardized, validated, and feasible definition is lacking. We tested the validity of a home-infusion CLABSI surveillance definition and the feasibility and acceptability of its implementation.

**Design:**

Mixed-methods study including validation of CLABSI cases and semistructured interviews with staff applying these approaches.

**Setting:**

This study was conducted in 5 large home-infusion agencies in a CLABSI prevention collaborative across 14 states and the District of Columbia.

**Participants:**

Staff performing home-infusion CLABSI surveillance.

**Methods:**

From May 2021 to May 2022, agencies implemented a home-infusion CLABSI surveillance definition, using 3 approaches to secondary bloodstream infections (BSIs): National Healthcare Safety Program (NHSN) criteria, modified NHSN criteria (only applying the 4 most common NHSN-defined secondary BSIs), and all home-infusion–onset bacteremia (HiOB). Data on all positive blood cultures were sent to an infection preventionist for validation. Surveillance staff underwent semistructured interviews focused on their perceptions of the definition 1 and 3–4 months after implementation.

**Results:**

Interrater reliability scores overall ranged from κ = 0.65 for the modified NHSN criteria to κ = 0.68 for the NHSN criteria to κ = 0.72 for the HiOB criteria. For the NHSN criteria, the agency-determined rate was 0.21 per 1,000 central-line (CL) days, and the validator-determined rate was 0.20 per 1,000 CL days. Overall, implementing a standardized definition was thought to be a positive change that would be generalizable and feasible though time-consuming and labor intensive.

**Conclusions:**

The home-infusion CLABSI surveillance definition was valid and feasible to implement.

Similar to patients in the acute-care setting, patients receiving home infusion therapy may be at risk of central-line–associated bloodstream infection (CLABSI). The extent of this burden has not been characterized because no surveillance definition for home-infusion–associated CLABSI has been validated.

In the acute-care setting, national policies have resulted in widely accepted CLABSI surveillance definitions^1–4^ that are reported through the National Healthcare Safety Network (NHSN).^2,5,6^ The implementation of CLABSI surveillance definitions, mandated reporting, and benchmarking has encouraged an emphasis on acute-care CLABSI prevention and had resulted in a 50% drop in the CLABSI standardized infection ratio (SIR) prior to the COVID-19 pandemic.^7–10^

For patients on home infusion therapy, no standardized CLABSI definition has been validated. A 2008 home-healthcare–associated bloodstream infection (BSI) definition developed by the Association for Professionals in Infection Control/Healthcare Infection Control Practices Advisory Committee^11^ relied on NHSN acute-care CLABSI surveillance criteria in use at the time.^2^ Adoption has been limited in part^12^ because it lacks essential components contained in the current NHSN acute-care CLABSI definition,^13^ such as how to distinguish a BSI from a common commensal and denominator criteria.^14,15^ Experts vary nationally in how they define home-infusion CLABSIs, particularly the optimal numerator, denominator, and inclusion and exclusion criteria.^16^ Initiatives through the National Home Infusion Association (NHIA), the American Society for Parenteral and Enteral Nutrition (ASPEN), and the home-health Outcome and Assessment Information Set (OASIS) encourage reporting of home-infusion complications but do not specifically define CLABSI.^17–20^ In earlier work, we convened a team of experts to create a surveillance definition for CLABSI in home infusion therapy,^21^ and we identified barriers and mitigating strategies to home-infusion CLABSI surveillance.^22,23^ The resulting definition required validation prior to wider use.

Gathering sufficient data to determine whether a BSI meets criteria for a secondary BSI may be particularly difficult in home infusion therapy because it requires access to information from unaffiliated hospitals.^23^ Hospital-onset bacteremia and fungemia (HOB)—any BSI ≥48 hours after hospital admission—may better distinguish hospital-level performance,^24,25^ and hospitals may soon report HOB.^26^ Earlier work did not characterize optimal approaches to secondary BSI in home-infusion CLABSI surveillance.^21^

The objective of this work was (1) to demonstrate the validity of a home-infusion CLABSI definition^21^ and (2) to determine surveillance staff perceptions of the feasibility and acceptability of the definition. Based on concerns about the potential difficulty of applying secondary BSI definitions in home infusion therapy, we also examined the impacts of 3 different approaches to handling secondary BSIs on definition validity.

## Methods

### Overall research approach

We performed a mixed-methods study including quantitative validation of the application of the CLABSI definition and qualitative semistructured interviews with home-infusion CLABSI surveillance staff focused on experiences with definition implementation. The study was approved by the Johns Hopkins Institutional Review Board.

### Setting

The Home Infusion CLABSI Prevention Collaborative (HICPC) is a collaborative of 5 large home-infusion agencies affiliated with academic medical centers. The agencies include 2 in the mid-Atlantic, 1 in the northeast, and 2 in the Midwest, and it covers portions of 14 states and Washington, DC. Also, 5 agencies implemented the definition, but due to institutional review board constraints, only 4 agencies submitted data for validation.

### Definition development and implementation

We used an expert-informed definition adapted from the acute-care NHSN CLABSI definition.^21,27^ Staff performing CLABSI surveillance at each agency participated in interactive monthly webinars focused on CLABSI surveillance and prevention and interacted with experts via videoconferences and emails. Staff attending the webinars were encouraged to present cases for discussion. In addition, we provided checklists and instructions for CLABSI surveillance.

Collaborative members began applying the CLABSI definition in May 2021. Based on feedback, we made minor modifications to the definition through July 2021. We applied changes retroactively (Table 1).^21^ We asked HICPC members to apply 3 different variations of the definition based on approaches to secondary BSIs: (1) secondary BSIs were excluded if they met NHSN criteria, referred to as “NHSN criteria”; (2) a subset of common secondary BSIs meeting NHSN criteria were excluded (ie, pneumonia or PNU2 or PNU3; urinary tract infection or SUTI1a, SUTI1b, or SUTI2; gastrointestinal infection or GIT2a, GIT2b, GIT2c, or GIT3b; and intraabdominal infection or IAB1, IAB2b, IAB3a, or IAB3b),^27^ referred to as “modified NHSN criteria”; or (3) no secondary BSIs were excluded, referred to as “home-infusion–onset bacteremia (HiOB) criteria.”

**Table 1.** Original and Final Definition for CLABSI in Home Infusion Therapy

Original Definition		Changed Definition	
**Inclusion criteria**			
• Had a CVC for >2 calendar days before the development of the BSI. • In-home care >48 hours. • A CVC that terminates at or close to the heart or in one of the great vessels that is used for infusion, blood withdrawal, or hemodynamic monitoring. • Anyone in whom home-infusion staff accessed an implanted port or CVC. • Include CVC even if it has migrated. • Implanted ports accessed within the last 72 hours. • A CVC that has been in place for >2 consecutive calendar days and following the first access of the CVC. • Anyone in whom staff taught the patient or caregivers how to self-manage the CVC. • Anyone in whom staff performed a CVC dressing or cap change. • Anyone in whom staff inserted a PICC. • Anyone in whom staff de-accessed an implanted port.		• In-home care for >2 calendar days. The first day of in-home care is considered either the calendar day after hospital or skilled nursing facility discharge if the patient is initiating or resuming home infusion therapy after a hospitalization or skilled nursing facility admission, or the first calendar day at home with a CVC if home infusion therapy is being initiated in the outpatient setting. AND • Had a CVC for >2 calendar days and following the first access of the CVC before the development of the BSI AND • A CVC that terminates at or close to the heart or in one of the great vessels that is used for infusion, blood withdrawal, or hemodynamic monitoring. Include this CVC even if it has migrated AND at least 1of the following: o Anyone in whom home-infusion staff accessed a CVC in the past month o Implanted ports accessed or de-accessed in calendar month by agency or affiliated staff, or supplies sent for infusion through port in the past month o Anyone in whom staff performed a CVC dressing or cap change in the past month o Anyone in whom staff inserted a PICC in the past month o Anyone who was seen in-person or via telemedicine for training in CVC care or clinical evaluation of the CVC in in the past month) o Anyone for whom supplies were provided in the past month, if the agency provides education or oversight to contracted home nursing agencies, or if the patient or caregiver has been determined by the agency to be independent in CVC care	

**NUMERATOR**			
Patient of Any Age	Patient <1 Year of Age	Patient of Any Age	Patient <1 Year Of Age
ONE of the following 2: 1. Recognized pathogen isolated from blood culture, AND pathogen is not related to infection at another site, OR 2. One of the following signs or symptoms: fever of 38°C (100.4°F), chills, or hypotension (systolic blood pressure ≤90 mm Hg), AND 1 of the following 2: (A) Common skin contaminant isolated from 2 blood cultures drawn on separate occasions (different venipunctures, a combination of venipuncture and lumen withdrawal, or different lumens of the same central line; or at different times) AND organism is not related to infection at another site, OR (B) Common skin contaminant isolated from blood culture from patient with intravascular access device AND provider institutes appropriate antimicrobial therapy (antimicrobial active against the organism initiated between 2 days prior to and 2 days after the blood culture).	Patient up to 1 year of age has at least 1 of the following: fever (≥38°C), hypothermia (<36°C), apnea, or bradycardia (heart rate <100 beats per minute), AND organism identified in blood is not related to an infection at another site AND the same common commensal is identified by a culture or nonculture based microbiological testing method from 2 or more blood specimens collected on separate occasions (different venipunctures, a combination of venipuncture and lumen withdrawal, or different lumens of the same central line, or at different times).	**Option 1** • Is not a common commensal per the NHSN common commensal list *AND (for NHSN criteria only)* • Pathogen identified in blood is not related to an infection at another site per NHSN criteria within 14 days prior to or 7 days after the blood culture collection. **Option 2** • Has the same common commensal isolated from 2 or more blood cultures drawn on separate occasions (different venipunctures, a combination of venipuncture and lumen withdrawal, or different lumens of the same central line; or at different times) within 24 hours AND • Has at least 1 of the following signs or symptoms: o Fever of >38°C (100.4°F), o Chills, o Hypotension (systolic blood pressure <90 mm Hg) AND • Pathogen identified in blood is not related to an infection at another site per NHSN criteria within 14 days prior to or 7 days after the blood culture collection.	**Option 1** • Is not a common commensal per the NHSN common commensal list *AND (for NHSN criteria only)* • Pathogen identified in blood is not related to an infection at another site per NHSN criteria within 14 days prior to or 7 days after the blood culture collection. **Option 2** • Has the same common commensal isolated from 2 or more blood cultures drawn on separate occasions (different venipunctures, a combination of venipuncture and lumen withdrawal, or different lumens of the same central line; or at different times) within 24 hours *AND* • Has at least 1 of the following signs or symptoms: o Fever of >38°C (100.4°F), o hypothermia (<36°C), o apnea, o tachypnea, o bradycardia (heart rate <100 beats per minute)], AND • Pathogen identified in blood is not related to an infection at another site per NHSN criteria within 14 days prior to or 7 days after the blood culture collection.
**Denominator**			
Day of admission to home infusion services with a CVC to the day of CVC removal, subtracting time spent in acute care hospitals.		Day of admission to home infusion services with a CVC to the day of CVC removal. The first day in home care is considered either of the following: 1. The calendar day after hospital or skilled nursing facility discharge if the patient is initiating or resuming home infusion therapy after a hospitalization or skilled nursing facility admission OR 2. The first calendar day at home with a CVC if home infusion therapy is being initiated in the outpatient setting. Denominator data are submitted per calendar month. Therefore, to be included in the denominator, the patient had to be an active home infusion patient in that month. Patients can be an active home-infusion patient by having at least 1 of the following: o Anyone in whom home infusion staff accessed a CVC. o Implanted ports accessed or de-accessed in calendar month by agency or affiliated staff, or supplies sent for infusion through port. o Anyone in whom staff performed a CVC dressing or cap change. o Anyone in whom staff inserted a PICC. o Anyone who was seen in-person or via telemedicine for training in CVC care or clinical evaluation of the CVC. o Anyone for whom supplies were provided, if the agency provides education or oversight to contracted home nursing agencies, or if the patient or caregiver has been determined by the agency to be independent in CVC care.	
**Exclusion criteria**			
• Hospital readmission within 2 days of hospital discharge • Midlines or peripheral venous catheter • Patients with a ventricular assistance device • Munchausen syndrome (or by proxy, known or suspected) • Hemodialysis catheters		• Hospital readmission within 2 calendar days of hospital discharge. • **If readmitted, first positive blood culture drawn on third or later hospital day** • Midlines or peripheral venous catheters • Plasmapheresis catheter • Hemodialysis catheter • Patients with ventricular assist device • Munchausen syndrome (or by proxy, known or suspected). • Epidermolysis bullosa • Has had bacteremia with at least 1 of the same organism within the last 14 days.	

Note. CVC, central venous catheter; PICC, peripherally inserted central catheter; NHSN, National Healthcare Safety Network.

### Validation of CLABSI surveillance

Because the central study team did not have access to the electronic health record (EHR) systems of all agencies or admitting hospitals, each agency was asked to upload all data they would use to make a CLABSI determination for each patient with positive blood cultures. Such information included emergency department notes, history and physical, signs and symptoms, progress notes, procedure notes, discharge summaries, pathology reports, radiology reports, and microbiology reports. In addition, each agency was asked whether the case met either of the 3 CLABSI definition variations, and if so, to describe which of the 3 criteria they met or provide information about secondary BSI criteria. Additional data were requested on patient demographics, central-line information, and other clinical data. Denominator data were also requested. Data were requested on a monthly basis from May 2021 through June 2022. An experienced IP (K.W.) blinded to the agency’s CLABSI determination reviewed all submitted data and determined whether the case would meet any or all of the CLABSI criteria. All positive blood cultures were presented for review.

### Analysis of CLABSI data

We first calculated CLABSI rates per 1,000 home-infusion central-line (CL) days. Data were calculated per agency overall, per agency over time, over all agencies, over all agencies over time, for the 3 definition variations, and as ascertained by both the agency and the IP validator. Descriptive statistics described the CLABSI rates for each agency.

We calculated interrater reliability (IRR) using the κ (kappa) statistic, comparing CLABSIs as ascertained by each agency and as ascertained by the IP validator. IRR was calculated for each of the 3 definitions, and separately for each agency. Because of concern that data might be asymmetric, we also calculated percentile agreement.^28^ Sensitivity and specificity were calculated using all submitted cases with bacteremia and the validator CLABSI determination.^29^

### Qualitative interview procedures

We constructed a semistructured interview guide focused on the home-infusion CLABSI surveillance definition’s feasibility, adoption, maintenance, acceptability, appropriateness, costs, and implementation strategies.^30^ We used purposive sampling to recruit semistructured interview participants.^31^ We started by purposively asking 1–4 staff members for each of the 5 agencies engaged in CLABSI surveillance to participate. We attempted to interview each eligible staff member twice after the May 2021 definition implementation: 1 month after using the definition and again 3–4 months after using the definition. After obtaining written consent, all interviews were conducted remotely via videoconferencing and lasted between 20 and 60 minutes. Interviews were audio-recorded and transcribed. We modified the definition based on findings from the first set of interviews and discussions in webinars.

### Qualitative data analysis

At the end of each interview, we sent audio files of recorded interviews to a licensed transcriptionist. Transcripts were deidentified and uploaded into MAXQDA for qualitative data management and analyses (VERBI Software, Berlin, GA). The initial codebook was developed deductively from the interview guide. The interview guide examined feasibility, adoption, maintenance, acceptability, appropriateness, costs,^30^ and implementation strategies. Deductive codes were applied to the first 3 interview transcripts. In addition, we inductively identified emergent subcodes to each parent code. This initial coding process was conducted by S.H. and S.C.K. Disagreements in coding were rectified to coder agreement.^32^ S.H. then coded the remainder of the data. S.H. and S.C.K. discussed emergent findings and modifications to the coding framework (Supplementary Material online). We engaged in a process of constant comparison of emergent findings throughout the analysis and when no new codes could be identified, we considered thematic saturation to have been achieved.^33^

## Results

### Validation

Agencies reported information on all patients with positive blood cultures, including 93 positive blood cultures for agency 1; 51 patients with positive blood cultures for agency 2; 60 patients with positive blood cultures for agency 3; and 40 patients with positive blood cultures for agency 4.

IRR, percent agreement, and sensitivity and specificity are liste in Table 2. IRR overall ranged from κ = 0.66 and 86.6% agreement for the modified NHSN criteria, to κ = 0.68 and 86.2% agreement for the NHSN criteria, to κ = 0.72 and 91.5% agreement for the HiOB criteria (Table 2). IRR varied between agencies, as did the definition variant for which agencies had the highest IRR. For agency 1, the HiOB variation was the most reliable; for agency 2, the modified NHSN variation was the most reliable; and for agency 3, the HiOB variation was the most reliable. Some reasons for discrepancies in CLABSI or HiOB classification included erroneous application of mucosal barrier injury criteria (due to anticipated difficulties in accessing data on neutropenia or diarrhea volume, mucosal barrier injury criteria was not included in the definition)^21^ and becoming accustomed to applying the definition.

**Table 2.** Interrater reliability, Sensitivity, Specificity, and Percent Agreement of Surveillance Staff of Home Infusion Agencies when Compared with Validator, for Each of Three Approaches to Handling Secondary Bloodstream Infections

Agency	NHSN Criteria			Modified NHSN Criteria			Home Infusion Onset Bacteremia Criteria		
	Interrater Reliability (95% CI)	Sensitivity (Specificity)	% Agreement	Interrater Reliability (95% CI)	Sensitivity (Specificity)	% Agreement	Interrater Reliability (95% CI)	Sensitivity (Specificity)	% Agreement
Agency 1	0.53 (0.35–0.70)	0.78 (0.79)	78.5	0.51 (0.33–0.70)	0.82 (0.73)	79.4	0.61 (0.39–0.83)	0.92 (0.77)	90.0
Agency 2	0.83 (0.67–0.99)	0.98 (0.82)	92.2	0.85 (0.68–1.00)	0.98 (0.85)	94.1	0.74 (0.46–1.00)	0.96 (0.83)	94.1
Agency 3	0.89 (0.77–1.00)	0.97 (0.91)	95.0	0.85 (0.70–0.99)	0.98 (0.85)	93.3	0.92 (0.81–1.00)	1.00 (0.90)	96.7
Agency 4	0.33 (0.00–0.72)	0.86 (0.67)	84.6	0.33 (0.00–0.72)	0.86 (0.67)	84.6	0.33 (0.00–0.72)	0.86 (0.67)	84.6
Overall	0.68 (0.58–0.78)	0.88 (0.83)	86.2	0.66 (0.56–0.77)	0.90 (0.79)	86.6	0.72 (0.61–0.83)	0.93 (0.83)	91.5

Note. CI, confidence interval; NHSN, National Health Safety Network.

**Fig. 1.**
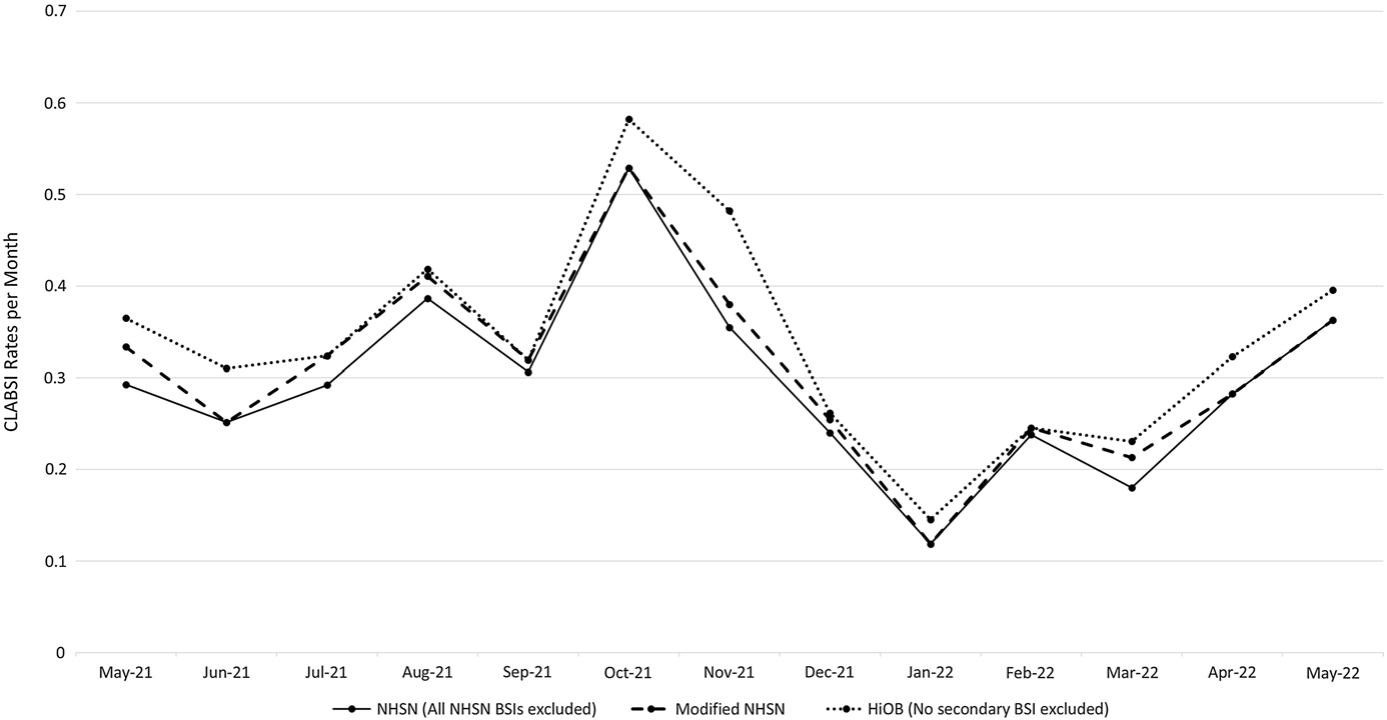
Central-line–associated bloodstream infections (CLABSI) rates per month per 1,000 central venous catheter days based on rates as determined by the single central-study team validator. Rates are reported across all agencies based on the full National Healthcare Safety Network (NHSN) criteria, modified NHSN criteria, and home infusion onset bacteremia (HiOB) criteria.

We also calculated CLABSI rates per 1,000 CL days (Table 3). For the NHSN criteria, the validator-determined rate was 0.20 per 1,000 CL days. For the modified NHSN criteria, the validator-determined rate was 0.21 per 1,000 CL days. For the HiOB criteria overall, the validator-determined rate was 0.23 per 1,000 CL days. Agency monthly CLABSI rates are reported for each variation definition (Fig. 1).

**Table 3.** CLABSIs per 1,000 Central-Line Days and Total CLABSIs per Home Infusion Agency Surveillance Staff and Per the Validator, for Each of the Three Approaches to Secondary Bloodstream Infections

Definition	NHSN Criteria Rate per 1,000 CL days (Total CLABSIs)		Modified NHSN Criteria Rate per 1,000 CL Days (Total CLABSIs)		Home Infusion Onset Bacteremia Criteria Rate per 1,000 CL Days (Total CLABSIs)	
	Agency	Validator	Agency	Validator	Agency	Validator
Agency 1	0.53 (62)	0.57 (67)	0.56 (65)	0.61 (71)	0.72 (84)	0.69 (80)
Agency 2	0.10 (41)	0.09 (39)	0.11 (40)	0.10 (44)	0.12 (45)	0.12 (51)
Agency 3	0.34 (39)	0.29 (36)	0.34 (42)	0.29 (39)	0.34 (46)	0.29 (43)
Agency 4	0.19 (39)	0.17 (39)	0.20 (39)	0.19 (33)	0.22 (33)	0.21 (33)
Overall	0.21 (181)	0.20 (175)	0.22 (186)	0.22 (187)	0.25 (214)	0.24 (207)

Note. CLABSI, central-line–associated bloodstream infection; CL, central line; NHSN, National Health Safety Network.

### Qualitative interviews

We interviewed HICPC members from the 5 agencies: 6 participants were interviewed 1 month after definition implementation and 8 participants were interviewed 3 months after implementation (Table 4). We identified 7 themes: (1) seeing the definition as a positive change, (2) identifying that the definition brought challenges, (3) seeing the definition as generalizable, (4) seeing the definition as feasible, (5) strategies for implementation, (6) lessons learned, and (7) considerations for applying different variants of the definition (Table 5).

**Table 4.** Characteristics of Home Infusion Agency Staff Participating in Semistructured Interviews 1 Month and 3–4 Months After Implementation of the Home Infusion CLABSI Surveillance Definition

Characteristics	1 Month After Implementation (N=6), No. (%)	3–4 Months After Implementation (N=8), No. (%)
**Home-infusion agency**		
Agency 1	1 (17)	1 (13)
Agency 2	2 (33)	2 (25)
Agency 3	2 (33)	3 (38)
Agency 4	1 (17)	1 (13)
Agency 5	0 (0)	1 (13)
Sex, female	6 (100)	8 (100)
**Race**		
Native American or Alaska Native	0 (0)	1 (13)
Black	0 (0)	0 (0)
White	6 (100)	7 (88)
Ethnicity, non-Hispanic	6 (100)	7 (88)
**Roles**		
Nurses	4 (67)	5 (63)
Pharmacists	0 (0)	1 (13)
Infection preventionists	2 (33)	2 (25)

Note. CLABSI, central-line–associated bloodstream infection.

**Table 5.** Perceptions of the Definition and Its Implementation

Theme	Subtheme	Illustrative Quote
Definition as a positive change	Improving over time	“I thought we had a solid definition to start with, but as we clarify things, it seems more of a solid definition, the more that we dig into it and work out the kinks in it, the more it feels like I know exactly what I’m looking for.” (Agency 3 infection preventionist)
	Using definition to provide feedback	“I think once it’s determined, like, yes, this is, it fits every piece of the definition. There’s even been times where I’ve requested that some documentation tips go back to the physicians. How successfully that gets through, I’m not sure, but there’s times where the notes are about as vague as they can be.” (Agency 4 nurse)
	Specificity	“So the CLABSI definition is easier to use. I like the fact that the transfer day is the first day. That has been obviously easier to kind of count back instead of trying to figure out what day back, so that definitely has helped. Like I said, the CLABSI definition in general has been fairly easy to use.” (Agency 3 nurse)
	Positive outcomes for patients	“There’s just going to be such a benefit [for patients]. I’m just so anxious to see where we are in 5 years when … all of our research is completed.” (Agency 2 nurse)
	Positive changes in workflow	“I think it really does make it easier to do this work, so I’m really thankful and grateful for it.” (Agency 3 nurse)
	Negligible impact on current workflow	“So there definitely has been an impact like in switching up the definition and kind of getting set, but the work that we’re doing already, it’s not extra work to put on what we were already doing. It’s just pulling a couple additional pieces of information to put into that report.” (Agency 3 infection preventionist)
	Clarify outcomes with leadership	“And then when we would give the data to executives, they would look at us and say, ‘Well, what does this mean,’ and then we were in this very horrible situation of, ‘Well, this is what it means for us but I can’t tell you what this means to, compared to acute care and I can’t tell you what it means compared to other infusion companies because I can’t guarantee you that we’re all measuring this the same way.’ So then they’re frustrated again.” (Agency 1 infection preventionist)
Definition does bring some challenges	Time consuming and labor intensive	“But no, that’s the hardest part, honestly, is just pulling, extracting all that data from the chart, especially if it’s a case where you don’t have as much access to the chart or the chart looks different because it’s not in Epic, you know, it’s a different kind of charting you’re going through.” (Agency 1 infection preventionist)
	Requires access to many computer systems	“Well, we’re switching to a new EMR by the end of the year… it might make a slight difference [but] I’m still going to have to individually go through and read notes, because there are no fields that I can pull a report from. … [we] have no way to do that outside of our EMR, so anytime I’ve got to go into the 4 different Epics I still have to look through the progress notes.” (Agency 4 nurse)
	Complicated definition	“I’m like going down a rabbit hole that I shouldn’t be going down, because if I go back to the definition it’s not something that’s pertinent.” (Agency 4 nurse)
	Does not address all cases	“Anytime you’re scratching your head it’s because of the patient’s situation.” (Agency 4 nurse)
	Need for workflow changes	“It has taken time for us to get into the groove with this, but … the other thing that has taken even additional time for us is just because of the computer difficulties that we had there, with our other nurse, so I think now that we have that straightened out, we’ll find that it will be much more streamlined. (Agency 2 nurse)
	Relying on others’ determination	“We’re basically being told that the patient has a CLABSI and the hospital systems are … basically using the definition … to define if the patient has a CLABSI and then they just tell us if they have it…. the hospital makes that diagnosis. We don’t do that.” (Agency 5 pharmacist)
Generalizable	Can be used by other agencies	“I think that other agencies, you know, potentially would benefit from using the definition.” (Agency 2 nurse)
	Requires training and practice	“I think [other agencies] could [use the definition] with education and with getting to know it. Like I said, it takes a moment to learn, just like anything, and then you become familiar with it and then put it into practice and then become proficient at it, and then it just, then you streamline it and then it doesn’t take as much time.” (Agency 3 infection preventionist)
	Requires organizational buy-in	“I think a lot of companies, what you’d say CLABSI and they’re just going to shake their head and say, ‘No, we’re not doing anything about that. We’re keeping track of infections, they’ll tell you, like raw infections, but we’re not defining them using any sort of standardized definition.’” (Agency 1 infection preventionist)
Feasible	Usable but secondary bloodstream infections are difficult	“The definition itself is usable. I think all the pieces that go into the work that goes, like behind the scenes, to determine if it’s actually a CLABSI or not is the piece that’s in question, and it’s different everywhere.” (Agency 4 nurse)
	Easier over time	“I do think that as we get more proficient with it, it will come easier, so as we’re able to feel more comfortable with the secondary BSI, just like, you know, when we first started doing CLABSI definition, it was a little time consuming, and then as we became more proficient it took less time. “ (Agency 3 nurse)
Strategies for implementation	Have definition readily accessible or as a checklist	“I think when you have a lot of them that you’re doing it’s easy to I think forget to like go through the actual pieces of it to make sure, but I just keep the definition in front of me to make sure that I’m not drifting anywhere.” (Agency 4 nurse)
Lessons learned from surveillance	Able to use data to learn about CLABSIs	“We do collect a little bit extra data just because we’re trying to identify if there’s any trends…. And then we also, I’m just pulling it up again, length of hospital stay was one of the things that we’re tracking as well, so if we do count them as a CLABSI, because we’re part of a larger health system, it’s really helpful to talk about quality initiatives and potential cost savings if we talk about length of hospital stay with leadership…. We also capture the number of catheter lumens, so we have catheter type but then the number of lumens, so once again to see if there is a higher incidence in different types of or catheters with multiple lumens….” (Agency 3 nurse)
Considerations for applying different variants of the definition	NHSN criteria precise and actionable but time-consuming and difficult to learn	“If we’re looking at actually improving the outcomes for patients… I feel like we have to keep [the secondary BSI criteria] broader.” (Agency 4 nurse) “[It requires] more work, a lot more … digging for information, definitely.” (Agency 2 nurse) “Secondary BSI, this is where I probably struggle the most, okay, so this is where there’s that link to a PDF manual of NHSN, and I have pulled that up, but I think that’s another like 500-some-page document where I’m going, I just circled it and put a big question mark there.” (Agency 3 nurse)
	Modified NHSN criteria identified most CLABSIs	“I think [the modified NHSN criteria is] still good to capture the secondary infections, especially in certain patient populations.” (Agency 3 infection preventionist)
	HiOB may be more efficient and replicable, but may artificially inflate rates, and may still take significant time	“And we certainly would not want the numbers to look like they’re elevated if we had to report… secondaries out … to the Board.” (Agency 2 infection preventionist)
Theme	Subtheme	Illustrative Quote
Definition as a positive change	Improving over time	“I thought we had a solid definition to start with, but as we clarify things, it seems more of a solid definition, the more that we dig into it and work out the kinks in it, the more it feels like I know exactly what I’m looking for.” (Agency 3 infection preventionist)
	Using definition to provide feedback	“I think once it’s determined, like, yes, this is, it fits every piece of the definition. There’s even been times where I’ve requested that some documentation tips go back to the physicians. How successfully that gets through, I’m not sure, but there’s times where the notes are about as vague as they can be.” (Agency 4 nurse)
	Specificity	“So the CLABSI definition is easier to use. I like the fact that the transfer day is the first day. That has been obviously easier to kind of count back instead of trying to figure out what day back, so that definitely has helped. Like I said, the CLABSI definition in general has been fairly easy to use.” (Agency 3 nurse)
	Positive outcomes for patients	“There’s just going to be such a benefit [for patients]. I’m just so anxious to see where we are in 5 years when … all of our research is completed.” (Agency 2 nurse)
	Positive changes in workflow	“I think it really does make it easier to do this work, so I’m really thankful and grateful for it.” (Agency 3 nurse)
	Negligible impact on current workflow	“So there definitely has been an impact like in switching up the definition and kind of getting set, but the work that we’re doing already, it’s not extra work to put on what we were already doing. It’s just pulling a couple additional pieces of information to put into that report.” (Agency 3 infection preventionist)
	Clarify outcomes with leadership	“And then when we would give the data to executives, they would look at us and say, ‘Well, what does this mean,’ and then we were in this very horrible situation of, ‘Well, this is what it means for us but I can’t tell you what this means to, compared to acute care and I can’t tell you what it means compared to other infusion companies because I can’t guarantee you that we’re all measuring this the same way.’ So then they’re frustrated again.” (Agency 1 infection preventionist)
Definition does bring some challenges	Time consuming and labor intensive	“But no, that’s the hardest part, honestly, is just pulling, extracting all that data from the chart, especially if it’s a case where you don’t have as much access to the chart or the chart looks different because it’s not in Epic, you know, it’s a different kind of charting you’re going through.” (Agency 1 infection preventionist)
	Requires access to many computer systems	“Well, we’re switching to a new EMR by the end of the year… it might make a slight difference [but] I’m still going to have to individually go through and read notes, because there are no fields that I can pull a report from. … [we] have no way to do that outside of our EMR, so anytime I’ve got to go into the 4 different Epics I still have to look through the progress notes.” (Agency 4 nurse)
	Complicated definition	“I’m like going down a rabbit hole that I shouldn’t be going down, because if I go back to the definition it’s not something that’s pertinent.” (Agency 4 nurse)
	Does not address all cases	“Anytime you’re scratching your head it’s because of the patient’s situation.” (Agency 4 nurse)
	Need for workflow changes	“It has taken time for us to get into the groove with this, but … the other thing that has taken even additional time for us is just because of the computer difficulties that we had there, with our other nurse, so I think now that we have that straightened out, we’ll find that it will be much more streamlined. (Agency 2 nurse)
	Relying on others’ determination	“We’re basically being told that the patient has a CLABSI and the hospital systems are … basically using the definition … to define if the patient has a CLABSI and then they just tell us if they have it…. the hospital makes that diagnosis. We don’t do that.” (Agency 5 pharmacist)
Generalizable	Can be used by other agencies	“I think that other agencies, you know, potentially would benefit from using the definition.” (Agency 2 nurse)
	Other agencies would also need training and practice	“I think [other agencies] could [use the definition] with education and with getting to know it. Like I said, it takes a moment to learn, just like anything, and then you become familiar with it and then put it into practice and then become proficient at it, and then it just, then you streamline it and then it doesn’t take as much time.” (Agency 3 infection preventionist)
	Other agencies would also need organizational buy-in	“I think a lot of companies, what you’d say CLABSI and they’re just going to shake their head and say, ‘No, we’re not doing anything about that. We’re keeping track of infections, they’ll tell you, like raw infections, but we’re not defining them using any sort of standardized definition.’” (Agency 1 infection preventionist)
Feasible	Usable but secondary bloodstream infections are difficult	“The definition itself is usable. I think all the pieces that go into the work that goes, like behind the scenes, to determine if it’s actually a CLABSI or not is the piece that’s in question, and it’s different everywhere.” (Agency 4 nurse)
	Easier over time	“I do think that as we get more proficient with it, it will come easier, so as we’re able to feel more comfortable with the secondary BSI, just like, you know, when we first started doing CLABSI definition, it was a little time consuming, and then as we became more proficient it took less time. “ (Agency 3 nurse)
Strategies for implementation	Have definition readily accessible or as a checklist	“I think when you have a lot of them that you’re doing it’s easy to I think forget to like go through the actual pieces of it to make sure, but I just keep the definition in front of me to make sure that I’m not drifting anywhere.” (Agency 4 nurse)
Lessons learned from surveillance	Able to use data to learn about CLABSIs	“We do collect a little bit extra data just because we’re trying to identify if there’s any trends…. And then we also, I’m just pulling it up again, length of hospital stay was one of the things that we’re tracking as well, so if we do count them as a CLABSI, because we’re part of a larger health system, it’s really helpful to talk about quality initiatives and potential cost savings if we talk about length of hospital stay with leadership…. We also capture the number of catheter lumens, so we have catheter type but then the number of lumens, so once again to see if there is a higher incidence in different types of or catheters with multiple lumens….” (Agency 3 nurse)
Considerations for applying different variants of the definition	NHSN criteria precise and actionable but time-consuming and difficult to learn	“If we’re looking at actually improving the outcomes for patients… I feel like we have to keep [the secondary BSI criteria] broader.” (Agency 4 nurse) “[It requires] more work, a lot more … digging for information, definitely.” (Agency 2 nurse) “Secondary BSI, this is where I probably struggle the most, okay, so this is where there’s that link to a.pdf manual of NHSN, and I have pulled that up, but I think that’s another like 500-some-page document where I’m going, I just circled it and put a big question mark there.” (Agency 3 nurse)
	Modified NHSN criteria identified most CLABSIs	“I think [the modified NHSN criteria is] still good to capture the secondary infections, especially in certain patient populations.” (Agency 3 infection preventionist)
	HiOB may be more efficient and replicable, but may artificially inflate rates, and may still take significant time	“And we certainly would not want the numbers to look like they’re elevated if we had to report… secondaries out … to the Board.” (Agency 2 infection preventionist)

Note. BSI, bloodstream infection; CLABSI, central-line–associated bloodstream infection; EMR, electronic medical record; HiOB, home-infusion–onset bacteremia; NHSN, National Health Safety Network.

Overall, participants saw the definition as a positive change. They observed that the definition improved over time and provided useful data for feedback. They noted that the definition was specific. They felt that it led to positive outcomes for patients, with negligible negative impact on workflow. It also helped them clarify outcomes with leadership. Participants did note that the definition brought challenges, including being time-consuming and labor intensive, requiring access to many EHRs, being complicated, not covering all patient situations, and relying on others’ determination.

Participants noted that the definition was generalizable. They felt that the definition could be used by other agencies, although it required training and practice as well as organizational buy-in. They also felt that the definition was feasible. They thought that the definition was usable, although understanding secondary BSIs was difficult. Strategies for implementation were suggested, particularly having the definition readily accessible or using a checklist when performing surveillance. Respondents also noted that they had learned lessons from CLABSI surveillance and could use data to learn about CLABSIs.

Respondents discussed consideration for applying the 3 definition variants. Overall, participants across agencies had positive perceptions of the NHSN criteria which they felt were precise and actionable but also felt that using secondary BSI criteria was time-consuming and difficult to learn. Meanwhile, respondents felt that the modified NHSN criteria identified most CLABSIs and was less time- and effort-intensive than the NHSN criteria. Finally, although some participants felt that the HiOB criteria were more efficient and replicable, they also felt that the HiOB criteria could inflate CLABSI rates. Others felt that using the HiOB definition would not save them as much time because they would still need to access and review similar amounts of data.

## Discussion

We worked with 5 large home-infusion agencies spanning parts of 14 states and the District of Columbia to refine, validate, and implement a surveillance definition for CLABSI in home infusion therapy. Overall, the reviewers showed good agreement with the trained IP using all 3 definition variations,^28,34^ although there were differences in how closely the agency staff and an IP agreed on the definition. The agency members saw the definition as a positive change, although it required time and training. Our research shows that the CLABSI definition was valid and that its implementation was feasible. The CLABSI rate of 0.21–0.23 per 1,000 CL days is lower than in acute-care settings, where the rate was 0.9 per 1,000 CL days in 2018.^35^

Application of the definition reached a “good” level of interrater reliability. The sensitivity and specificity shown here compared favorably with validations of inpatient data. Definitions of acute-care CLABSIs have shown sensitivities of 42%–88% and specificities of 70%–99%.^36^ Meanwhile, state health departments validating CLABSI reporting to the NHSN have reported a sensitivity of 83% and specificity of 99%.^37^ Sensitivity in our study ranged from 88% to 93%, while specificity in our study ranged from 79% to 83%. Overall, the validity of agency staff applying the home-infusion CLABSI definition was similar to these reports from acute care.

Participants felt that the NHSN criteria was more time-consuming and difficult to perform than the HiOB criteria, and we learned in initial webinars and early interviews with CLABSI surveillance staff^38^ that access to EHR-based data needed for application of NHSN secondary BSI criteria was incomplete and varied by site. There were no substantial differences between the CLABSI rates based on the 3 definition variations, which ranged from 0.20 to 0.23 per 1,000 CL days. Use of the HiOB criteria did not substantially inflate the CLABSI rate. IRRs ranged from 0.65 for the modified NHSN criteria, to 0.68 for the NHSN criteria, to 0.72 for the HiOB criteria. In acute care, hospitals may begin reporting HOB, similar to HiOB, as early as 2023.^26^ Data showed that although HOB data may be easily extracted from the EHR and HOB rates closely paralleled those of hospital CLABSIs,^24^ only 54% of hospital epidemiologists viewed HOB as reflecting quality. Many hospital epidemiologists preferred reporting both CLABSIs and HOB.^39^ HICPC members reviewing these data felt that the NHSN criteria made their CLABSI reporting more actionable and was important for agency quality and safety initiatives. Therefore, they requested that they continue to report both the NHSN criteria and HiOB criteria.

We examined perceptions of the definition. Overall, participants viewed the definition as a positive change with some challenges. They felt that it was generalizable and feasible. They provided strategies for implementation such as having a definition readily accessible or as a checklist (similar to that available for acute-care settings^40^). Finally, they provided considerations for applying different variants of the definition.

Agencies had prior experience in CLABSI surveillance, but earlier work noted barriers to home-infusion CLABSI surveillance. Barriers included having complicated tasks, the need for education in surveillance tailored to this setting, the need for assistance from information technologists and data analysts, the requirement for organizational support, and the need to manage information and communication.^22,23^ Therefore, we supported and guided agencies through approaches such as providing educational resources, suggestions on how to work with information technology, and interactive webinars. Providing education, enabling communication, and ensuring EHR support are essential.

Our research had several limitations. The agencies involved in the study were interested in CLABSI surveillance and may not represent agencies nationally. Due to ownership struggles, 1 participating home-infusion agency could not fully undergo approval from their institutional review board. Therefore, although 5 agencies participated in the qualitative interviews and the implementation of the definition, only 4 agencies participated in the validation of the definition. Due to ownership changes, changes in EHR vendors, difficulties accessing appropriate data, and staffing constraints related to the COVID-19 pandemic, some agencies had more challenges with data submission than others, although all were asked to initiate the definition at the same time.

We also depended on 1 trained IP to serve as the validator, but cases were frequently discussed with multiple members of the team. We depended on agencies to submit chart data to the central study team for use in validation. The central study team did not have access to the full EHR of all hospitals to which patients could be admitted (even individual home infusion agencies did not always have access to the EHRs of all hospitals to which patients could be admitted).^23^ Therefore, we were unable to ascertain whether agencies had truly submitted all relevant data. However, in this real-world study, the agencies provided us with access to the same data they could access. Agencies may not have known whether there were relevant data (eg, an unavailable progress note) that could help in making their determination. Future work should examine approaches to address data that were missing but not at random^41^ and the future impact of changing access to data.

The surveillance staff at each agency required extensive training and support in application of the definition, which may limit generalizability. We recently described the need for ongoing education in those performing home-infusion surveillance,^22^ such as through modification of pre-existing resources through the CDC and national organizations, development of new resources, or peer-learning collaboratives, and in training in accessing data. Finally, in home infusion therapy, patients with BSIs are typically cared for in hospitals and not in the home, so the agencies themselves were infrequently involved in blood-culture decisions. Hospitals to which home-infusion patients may be admitted may differ in their blood-culture practices.

We performed the first validation study of a home-infusion CLABSI surveillance definition and found that the definition was valid, feasible, and acceptable. CLABSIs in this setting occur at a lower rate than in acute-care hospitals. In addition, differences between using an NHSN approach to secondary BSIs and an HiOB approach had minimal impact on infection rates or validity, suggesting that each of these approaches may be acceptable and that agencies may choose which approach works best for their setting. System-level supports, such as training in surveillance and better integration of EHRs, would be helpful. Additional research should investigate acceptability of the definitions among other stakeholders (eg, leadership or frontline staff) as well as larger-scale validation of the definitions and use this definition to test CLABSI prevention interventions. National implementation of the home-infusion CLABSI definition would provide critical surveillance data to motivate and inform efforts to prevent CLABSIs in home infusion therapy.

## Supplementary material

For supplementary material accompanying this paper visit https://doi.org/10.1017/ice.2023.70. click here to view supplementary material
