# Supplementary material for: Implementing and validating a home-infusion central-line–associated bloodstream infection surveillance definition
Source: Infect Control Hosp Epidemiol. 2023 Apr 20;44(11):1748–59. doi: 10.1017/ice.2023.70 (PMC10665867; doi:10.1017/ice.2023.70)
Supplement: Supplementary file 1 [file S0899823X23000703sup001.docx]

**Supplemental Material: Codebook Used for Qualitative Interviews**

| **Domains / Parent Codes** | **Areas of Assessment / Sub-codes** | **Definition** | **Relevant Question(s)** | **Prompt(s)** |
| --- | --- | --- | --- | --- |
| Definition Appropriateness / Usability |  |  |  |  |
|  | Definition appropriateness / Ease of use | Ease of use of the definition | Is the definition easy or hard to use? | What was the easiest part? What was the hardest part? |
|  | Definition appropriateness / Other agencies | Feasibility of other agencies using the definition | Do you think other home infusion agencies could use this definition? |  |
|  | Definition appropriateness / Actionability | Utility of the definition in leading to an action or a positive change | Do you think the definition is actionable? How or why not? | Accuracy?  Actionability of result? |
|  | Definition appropriateness / Fit | Closeness of the definition to what is actually desired, items that the definition may miss | Does the current definition miss anything? | If so, what? |
|  | Definition appropriateness / Changes | Desired changes to the definition | Would you make any changes to the definition? | Why would you make this change?  What impact might these changes have?  Do you think everyone could make this change? |
|  | Definition appropriateness / Workflow Compatibility | Ability of the definition to fit into the workflow, items that may help the definition better fit into the workflow | What would help it fit into your workflow better? |  |
| Maintenance |  |  |  |  |
|  | Opinion change | Change in thoughts or perceptions of the definition over time | Has your opinion on the definition changed? |  |
| Results of Reporting using the Definition | Approach / Time | Length of time taken to apply the definition for individual cases |  | Amount of time taken |
|  | Approach / Accuracy | Accuracy of definition for reporting |  | Accuracy |
|  | Approach / Actionability | Actionability of the results for reporting, utility of the definition in leading to an action or a positive change |  | Actionability of result |
|  | Approach / Preference | Overall preference for using the reporting results. |  |  |
| Variations | Impact / Time | Distinctions in time taken to apply the definition for each of the three variations in the definitions. | What impact would each of these have on CLABSI reports? | Amount of time taken |
|  | Impact / Accuracy | Describes accuracy and relevance of the definition for each of the three variations in the definitions. |  | Accuracy |
|  | Impact / Actionability | Describes utility of the definition in leading to an action or a positive change for each of the three variations in the definitions. |  | Actionability of result |
|  | Impact/preference | Wwhich is preferred of the three variations in the definitions? |  |  |
| Negative outcomes and Costs |  |  |  |  |
|  | Negative outcomes / Time | Costs in terms of time, general descriptions of how much time the definition application takes | What costs are there in applying the definition? | Amount of time takes  How much time it takes |
|  | Negative outcomes / Prioritization | Costs in terms of time that could have been spent doing other things, other projects or initiatives or tasks that were not performed because of applying the definition |  | Time taken from other projects |
|  | Negative outcomes / Data pulls | Costs in terms of needing work with information technology specialists, data analysts, etc; or descriptions of time they took. |  | Need to build data pulls in electronic health record system, etc. |
| Workflow changes |  | Overall descriptions of changes to general processes or workflow to implement the definition | Have you made any changes to your workflow due to the definition? | What were they? |
|  | Workflow changes / Positive | Overall descriptions of changes to general processes or workflow to implement the definition that were positive or helped |  | Were these positive changes? |
|  | Workflow changes/ Negative | Overall descriptions of changes to general processes or workflow to implement the definition that were negative or hindered work |  | Were these negative changes? |
| Acceptability |  | Overall acceptability of the definition |  |  |
|  | Implementation variants | Acceptability of each of the three definition variants, preference for either of the definition variants, recommendations to use each of the definition variants | Which of these variations would you recommend using at this point? |  |
| Adoption |  | Descriptions of whether others in the organization have started to use or been aware of the definition |  |  |
|  | Uptake of definition / Teaching | Descriptions of training other staff in the new definition, descriptions of educating staff about this process | Have you had to teach any other staff about the new definition?  What did you tell them?  How did you teach them? | If so, who (role)?  About why the definition was changing?  What did you teach them? |
|  | Uptake of definition / Communication with leadership | Descriptions of discussions with leadership or administration at the organization about the definition, or the process of validating the definition | Have you spoken to leadership about the new definition? | Tell me about that conversation. |
| Change from prior |  |  |  |  |
|  | Previous definition | Descriptions of prior definitions used in CLABSI surveillance or how they were used | What definition were you using before? |  |
|  | Differences | Descriptions of how the CLABSI definition being validated may differ from definitions in use before | What differences were there with what you were using before and what the collaborative started using? |  |
| Training in definition |  | Focus here is on how the interviewee was trained |  |  |
|  | Training in definition / How trained | Overall experience in training in the CLABSI definition, methods of disseminating training material, overall perceptions of training | How did you learn about the new CLABSI definition?  What was your experience like learning about the CLABSI definition? | Webinar, e-mail, communication with project team, etc. |
|  | Training in definition / Helpful | Portions of the training that were positive and helpful | What did you find most helpful? |  |
|  | Training in definition / Not helpful | Portions of the training that were less positive and less helpful | What did you find to be not as helpful? |  |
|  | Training in definition / Suggested changes | Suggested changes in how training was provided, such as modality or topics | What should have been done differently? |  |
| Uncertainty / Confusion |  | Specific areas of uncertainty with the definition |  |  |
| Suggestions |  | General advice for implementation of the definition |  |  |
|  | Suggestions / Suggested Changes | Suggested changes for implementation of the definition that do not need action now |  |  |
|  | Suggestions / Areas requiring action | Suggested changes for implementation of the definition that do need action |  |  |
